# Supplementary material for: Oxonium ion scanning mass spectrometry for large-scale plasma glycoproteomics
Source: Nat Biomed Eng. 2023 Jul 20;8(3):233–47. doi: 10.1038/s41551-023-01067-5 (PMC10963274; doi:10.1038/s41551-023-01067-5)
Supplement: Supplementary file 1 — Supplementary Tables and References. [file 41551_2023_1067_MOESM1_ESM.pdf]

nature biomedical engineering

Article https://doi.org/10.1038/s41551-023-01067-5

Oxonium ion scanning mass spectrometry for large-scale plasma glycoproteomics

In the format provided by the authors and unedited

Supplementary Table 1 l IgG glycopeptides identified by OxoScan-MS analysis.

IgG	Glycan	m/z	Monoisotopic Mass	Charge	RT	[M+H]+	Peptide Mass	Glycan Mass	peak_num	Literature Precursor Monoisotopic	PPM Mass Error
1	G0	830.0054	2486.9943	3	27.55	2488.0016	1188.5047	1299.4969	17	2486.981	5.484
1	G0F	878.6832	2633.0277	3	26.85	2634.035	1188.5047	1445.5303	7	2633.039	4.128
1	G0FN	946.3678	2836.0815	3	27.95	2837.0888	1188.5047	1648.5841	14	2836.118	12.849
1	G1F	932.6988	2795.0745	3	26.55	2796.0818	1188.5047	1607.5771	4	2795.091	6.044
1	G1F	932.7058	2795.0955	3	26.96	2796.1028	1188.5047	1607.5981	18	2795.091	1.47
1	G1FN	1000.3946	2998.1619	3	27.75	2999.1692	1188.5047	1810.6645	13	2998.171	2.957
1	G1FS1	1029.7458	3086.2155	3	28.75	3087.2228	1188.5047	1898.7181	21	3086.187	9.296
1	G2F	986.7129	2957.1168	3	26.46	2958.1241	1188.5047	1769.6194	8	2957.144	9.271
1	G2FN	1054.4152	3160.2237	3	27.55	3161.231	1188.5047	1972.7263	20	3160.224	0.035
1	G2FS1	1083.7589	3248.2548	3	28.15	3249.2621	1188.5047	2060.7574	11	3248.24	4.669
2	G0	819.3432	2455.0077	3	52.15	2456.015	1156.5149	1299.5001	35	2454.991	6.859
2	G0F	868.0212	2601.0417	3	50.95	2602.049	1156.5149	1445.5341	1	2601.049	2.718
2	G0FN	935.703	2804.0871	3	51.25	2805.0944	1156.5149	1648.5795	9	2804.128	14.636
2	G1F	922.0331	2763.0774	3	50.15	2764.0847	1156.5149	1607.5698	3	2763.102	8.756
2	G1F	922.031	2763.0711	3	50.75	2764.0784	1156.5149	1607.5635	5	2763.102	11.036
2	G1FN	989.7264	2966.1573	3	50.55	2967.1646	1156.5149	1810.6497	12	2966.181	7.978
2	G1FN	989.7217	2966.1432	3	50.95	2967.1505	1156.5149	1810.6356	27	2966.181	12.732
2	G1FS1	1019.0768	3054.2085	3	53.65	3055.2158	1156.5149	1898.7009	10	3054.197	3.762
2	G2F	976.0541	2925.1404	3	49.85	2926.1477	1156.5149	1769.6328	2	2925.154	4.792
2	G2FN	1043.7621	3128.2644	3	50.25	3129.2717	1156.5149	1972.7568	15	3128.234	9.785
2	G2FS1	1073.0854	3216.2343	3	52.75	3217.2416	1156.5149	2060.7267	6	3216.25	4.829
4	G0F	873.36	2617.0581	3	38.05	2618.0654	1172.5098	1445.5556	19	2617.044	5.514
4	G0FN	941.0292	2820.0657	3	38.55	2821.073	1172.5098	1648.5632	33	2820.123	20.333
4	G1F	927.3766	2779.1079	3	37.45	2780.1152	1172.5098	1607.6054	23	2779.096	4.105
4	G1F	927.3892	2779.1457	3	37.15	2780.153	1172.5098	1607.6432	69	2779.096	17.706
4	G1F	927.3757	2779.1052	3	37.95	2780.1125	1172.5098	1607.6027	43	2779.096	3.133
4	G1FN	995.058	2982.1521	3	38.15	2983.1594	1172.5098	1810.6496	36	2982.176	7.969
4	G2F	981.38	2941.1181	3	37.24	2942.1254	1172.5098	1769.6156	26	2941.149	10.614
4	G2FN	1049.0927	3144.2562	3	37.85	3145.2635	1172.5098	1972.7537	81	3144.229	8.75
4	G2FS1	1078.4419	3232.3038	3	39.55	3233.3111	1172.5098	2060.8013	24	3232.245	18.274

Supplementary Table 2 l COVID-19 clinical cohort demographics.

Sample ID	Age	Sex	WHO Grade	Clinical Category	Sample ID	Age	Sex	WHO Grade	Clinical Category
HD-1	27	w	0	Healthy	CV-66	75	w	3	Mild
HD-2	23	m	0	Healthy	CV-75	84	m	3	Mild
HD-3	34	w	0	Healthy	CV-11	61	m	5	Moderate
HD-4	31	w	0	Healthy	CV-24	64	m	4	Moderate
HD-5	30	m	0	Healthy	CV-38	70	m	4	Moderate
HD-6	41	w	0	Healthy	CV-48	48	m	4	Moderate
HD-7	24	w	0	Healthy	CV-50	78	m	4	Moderate
HD-8	27	m	0	Healthy	CV-16	74	m	5	Moderate
HD-9	30	w	0	Healthy	CV-63	61	m	5	Moderate
HD-10	34	w	0	Healthy	CV-8	63	m	7	Severe
HD-11	42	m	0	Healthy	CV-9	80	w	7	Severe
HD-12	30	w	0	Healthy	CV-13	71	m	7	Severe
HD-13	33	w	0	Healthy	CV-25	45	w	7	Severe
HD-14	29	w	0	Healthy	CV-32	50	w	7	Severe
HD-15	36	w	0	Healthy	CV-33	35	w	7	Severe
CV-1	22	m	3	Mild	CV-57	55	w	6	Severe
CV-19	52	m	3	Mild	CV-58	26	m	7	Severe
CV-23	44	w	3	Mild	CV-59	86	m	7	Severe
CV-35	37	w	3	Mild	CV-60	65	m	6	Severe
CV-37	78	m	3	Mild	CV-61	54	m	7	Severe
CV-49	72	w	3	Mild	CV-62	72	m	6	Severe
CV-56	58	w	3	Mild	CV-64	57	m	7	Severe
CV-65	22	m	3	Mild					

Supplementary Table 3 I Non-linear chromatographic gradient for COVID-19 cohort method.

Time	Flow rate (µl/min)	% A	% B
0.00	5	97.0	3.0
0.86	5	92.9	7.1
2.42	5	88.8	11.2
5.53	5	84.7	15.3
9.38	5	80.6	19.4
13.02	5	76.4	23.6
15.48	5	72.3	27.7
17.27	5	68.2	31.8
19.00	5	60.0	40.0
20.00	5	20.0	80.0
20.50	5	20.0	80.0
21.50	5	97.0	3.0
27.50	5	97.0	3.0

Supplementary Table 4 I Scanning SWATH parameters for different gradients. All chromatographic gradients are linear (3-40% Buffer B) except that described in Table S2.

Method Name	Active Gradient Length (min)	Precursor Range (m/z)	Window Width (m/z)	Cycle Time (s)	Effective Accumulation Time (ms)
Cohort	19	800–1400	10	1.5	21.7
Deglyco	45	1000–1500	5	3	28.2
IgG	90	800–1600	10	6	71.5
GPF-1	90	500–1000	1	6	11.7
GPF-2	90	1000–1500	1	6	11.7
GPF-3	90	1500–2000	1	6	11.7

Supplementary Table 5 I Glycopeptides identified in DDA experiments and quantified across the COVID-19 cohort by OxoScan-MS.

Genes	Peptide	Glycan Short	Position	Calc M+H	Observed m/z	z	peak_num
AHSG	K.VC[+57.021]QDC[+57.021]PLLAPLN[+2204.772]DTR.V	N4H5S2	N156	3976.619	1326.2104	3	98
AHSG	R.TVVQPS[+656.228]VGAAAGPVVPPC[+57.021]PGR.I	N1H1S1	S346	2672.2971	891.4379	3	66
FGB	R.GTAGNALMDGASQLMGEN[+1913.677]R.T	N4H5S1	N394	3806.5359	1903.7694	2	8
FGB	R.GTAGNALMDGASQLMGEN[+2204.772]R.T	N4H5S2	N394	4097.6314	1366.5474	3	11
HP	K.MVSHHN[+2204.772]LTTGATLINEQWLLTTAK.N	N4H5S2	N184	4884.1647	1628.7245	3	1
HP	K.MVSHHN[+2861.000]LTTGATLINEQWLLTTAK.N	N5H6S3	N184	5540.3924	1385.8518	4	23
HP	K.VVLHPN[+2204.772]YSQVDIGLIK.L	N4H5S2	N241	3999.7837	1333.9315	3	3
IGHA1	R.LAGKPTHVN[+2262.814]VSVVMAEVDGTC[+57.021].Y	N5H5S1F1	N340	4446.8931	1482.9686	3	172
IGHA1	R.LAGKPTHVN[+2350.830]VSVVMAEVDGTC[+57.021].Y	N4H5S2F1	N340	4534.9091	1512.3063	3	73
IGHA1	R.LAGKPTHVN[+2553.910]VSVVMAEVDGTC[+57.021].Y	N5H5S2F1	N340	4737.9885	1579.9986	3	50
IGHA1;IGHA2	R.LSLHRPALEDLLLGSEAN[+1216.423]LTC[+57.021]TLTGLR.D	N2H5	N144	4180.0211	1045.7597	4	130
IGHA1;IGHA2	R.LSLHRPALEDLLLGSEAN[+1501.555]LTC[+57.021]TLTGLR.D	N5H3	N144	4465.1536	1117.0431	4	28
IGHA1;IGHA2	R.LSLHRPALEDLLLGSEAN[+1751.624]LTC[+57.021]TLTGLR.D	N4H4S1	N144	4715.2225	1179.559	4	40
ORM1	K.WFYIASAFRNEEYN[+2204.772]K.S	N4H5S2	N56	4142.6905	1381.5667	3	142
SERPINA1	K.YLGN[+1913.677]ATAIFFLPDEGKLQHLENELTHDIITK.F	N4H5S1	N271	5454.5143	1364.6344	4	39
SERPINA1	R.QLAHQSN[+2204.772]STNIFFSPVSIATAFAMLSLGTK.A	N4H5S2	N70	5386.4075	1796.1399	3	5
TF	R.QQQHLFGSN[+2204.772]VTDC[+57.021]SGNFC[+57.021]LFR.S	N4H5S2	N630	4719.8966	1573.9675	3	12

Supplementary Table 6 | Glycopeptides, adjacent, unmodified and QC peptides monitored by MRM-HR acquisition.

Genes	Molecule	Precursor Charge	RT	m/z	Peptide Type
AHSG	K.VC[+57.021]QDC[+57.021]PLLAPLN[+2204.772]DTR.V	3	14.2	1326.243	Glycopeptide
AHSG	R.TVVQPS[+656.228]VGAAAGPVVPPC[+57.021]PGR.I	3	10.5	891.458	Glycopeptide
FGB	R.GTAGNALMDGASQLMGEN[+1622.582]R.T	3	14.6	1172.517	Glycopeptide
FGB	R.GTAGNALMDGASQLMGEN[+1913.677]R.T	3	14.6	1269.550	Glycopeptide
FGB	R.GTAGNALMDGASQLMGEN[+2204.772]R.T	3	14.8	1366.556	Glycopeptide
FGG	K.VDKDLQSLEDILHQVEN[+1622.582]K.T	4	17.2	937.209	Glycopeptide
FGG	K.VDKDLQSLEDILHQVEN[+1913.677]K.T	4	17.6	1009.979	Glycopeptide
HP	K.MVSHHN[+2204.772]LTTGATLINEQWLLTTAK.N	4	16.6	1221.832	Glycopeptide
HP	K.MVSHHN[+2350.830]LTTGATLINEQWLLTTAK.N	4	16.5	1258.352	Glycopeptide
HP	K.MVSHHN[+2861.000]LTTGATLINEQWLLTTAK.N	4	16.8	1385.894	Glycopeptide
HP	K.VVLHPN[+2204.772]YSQVDIGLIK.L	3	15.0	1333.968	Glycopeptide
HPX	R.SWPAVGN[+2204.772]C[+57.021]SSALR.W	3	11.0	1203.846	Glycopeptide
IGHA1	R.LAGKPTHVN[+2262.814]VSVVMAEVDGTC[+57.021].Y	4	14.2	1112.508	Glycopeptide
IGHA1	R.LAGKPTHVN[+2350.830]VSVVMAEVDGTC[+57.021].Y	4	14.6	1134.516	Glycopeptide
IGHA1	R.LAGKPTHVN[+2553.910]VSVVMAEVDGTC[+57.021].Y	4	14.7	1185.275	Glycopeptide
IGHA1;IGHA2	R.LSLHRPALEDLLLGSEAN[+1216.423]LTC[+57.021]TLTGLR.D	4	19.5	1045.797	Glycopeptide
IGHA1;IGHA2	R.LSLHRPALEDLLLGSEAN[+1501.555]LTC[+57.021]TLTGLR.D	4	19.4	1117.081	Glycopeptide
IGHA1;IGHA2	R.LSLHRPALEDLLLGSEAN[+1751.624]LTC[+57.021]TLTGLR.D	4	19.8	1179.601	Glycopeptide
IGHA1;IGHA2	R.LSLHRPALEDLLLGSEAN[+2116.756]LTC[+57.021]TLTGLR.D	4	19.7	1270.889	Glycopeptide
ORM1	K.WFYIASAFRNEEYN[+2204.772]K.S	4	16.7	1036.459	Glycopeptide
SERPINA1	K.YLGN[+1913.677]ATAIFFLPDEGKLQHLENELTHDIITK.F	5	19.8	1091.751	Glycopeptide
SERPINA1	K.YLGN[+2204.772]ATAIFFLPDEGK.L	3	18.4	1320.935	Glycopeptide
SERPINA1	R.QLAHQSN[+2204.772]STNIFFSPVSIATAFAMLSLGTK.A	4	21.0	1347.409	Glycopeptide
TF	R.QQQHLFGSN[+2204.772]VTDC[+57.021]SGNFC[+57.021]LFR.S	4	16.2	1180.763	Glycopeptide
A1BG	HQFLLTGDTQGR	3	9.2	458.238	QC
C3	AAVYHHFISDGVR	3	7.6	491.254	QC
C8G	SLPVSDSVLSGFEQR	3	16.4	540.946	QC
CLU	VTTVASHTSDSDVPSGVTEVVVK	3	19.1	772.065	QC
F2	VIDQFGE	1	9.7	807.389	QC
GC	THLPEVFLSK	2	12.8	585.830	QC
ITIH2	VQFELHYQEVK	3	12.3	473.914	QC
KNG1	TVGSDTFYSFK	2	13.0	626.299	QC
PLG	VILGAHQEVNLEPHVQEIEVSR	4	12.7	624.836	QC
SERPINC1	ATEDEGSEQKIPEATNR	3	17.5	625.630	QC
AHSG	TVVQPSVGAAAGPVVPPCPGR	3	11.1	672.695	Unmodified peptides
AHSG	TVVQPSVGAAAGPVVPPCPGR	2	11.1	1008.539	Unmodified peptides
HP	VVLHPNYSQVDIGLIK	2	14.7	898.009	Unmodified peptides
HP	VVLHPNYSQVDIGLIK	3	14.7	599.009	Unmodified peptides
HP	VVLHPNYSQVDIGLIK	4	14.7	449.508	Unmodified peptides
HPX	SWPAVGNCSSALR	2	11.4	702.838	Unmodified peptides
TF	QQQHLFGSNVTDCSGNFCLFR	3	16.3	839.046	Unmodified peptides

AHSG	CNLLAEK	2	7.4	424.221	Adjacent peptides
AHSG	EHAVEGDCDFQLLK	3	12.2	554.259	Adjacent peptides
AHSG	FSVVYAK	2	9.4	407.229	Adjacent peptides
HP	DYAEVGR	2	5.1	405.193	Adjacent peptides
HP	SCAVAEYGVYVK	2	10.8	673.326	Adjacent peptides
HP	TEGDGVYTLNDK	2	7.7	656.307	Adjacent peptides
HP	TEGDGVYTLNNEK	2	7.5	720.336	Adjacent peptides
HPX	GGYTLVSGYPK	2	10.7	571.298	Adjacent peptides
HPX	LYLVQGTQVYVFLTK	2	19.2	886.503	Adjacent peptides
HPX	SLGPNSCSANGPGLYLIHGPNLYCYSDVEK	4	16.9	821.385	Adjacent peptides
IGHA1	QEPSQGTTTFAVTSILR	3	16.7	612.655	Adjacent peptides
IGHA1	SAVQGPPER	2	4.3	470.746	Adjacent peptides
IGHA1	TFTCTAAYPESK	2	7.4	688.314	Adjacent peptides
IGHA1	TPLTATLSK	2	8.7	466.277	Adjacent peptides
IGHA1;IGHA2	NFPPSQDASGDLYTTSSQLTLPATQCPDGK	4	16.3	799.874	Adjacent peptides
IGHA2	QEPSQGTTTYAVTSILR	3	15.0	617.986	Adjacent peptides
ORM1	EQLGEFYEALDCLR	2	18.6	871.906	Adjacent peptides
ORM1	SDVVYTDWK	2	10.8	556.767	Adjacent peptides
ORM1	YVGGQEHFAHLLILR	3	14.5	584.990	Adjacent peptides
TF	CSTSSLLEACTFR	2	13.4	766.348	Adjacent peptides
TF	DDTVCLAK	2	6.3	461.221	Adjacent peptides
TF	IECVSAETTEDCIAK	2	8.2	863.387	Adjacent peptides
TF	YLGEEYVK	2	9.7	500.753	Adjacent peptides

References

1. Wieczorek, M., Braicu, E. I., Oliveira-Ferrer, L., Sehouli, J. & Blanchard, V. Immunoglobulin G Subclass-Specific Glycosylation Changes in Primary Epithelial Ovarian Cancer. *Front. Immunol.* **11**, 654 (2020).
2. Demichev, V., Messner, C. B., Vernardis, S. I., Lilley, K. S. & Ralser, M. DIA-NN: neural networks and interference correction enable deep proteome coverage in high throughput. *Nat. Methods* **17**, 41–44 (2020).
3. Ritchie, M. E. *et al.* limma powers differential expression analyses for RNA-sequencing and microarray studies. *Nucleic Acids Res.* **43**, e47 (2015).
4. Benjamini, Y. & Hochberg, Y. Controlling the False Discovery Rate: A Practical and Powerful Approach to Multiple Testing. *J. R. Stat. Soc. Series B Stat. Methodol.* **57**, 289–300 (1995).
