# Supplementary material for: Oxonium ion scanning mass spectrometry for large-scale plasma glycoproteomics
Source: Nat Biomed Eng. 2023 Jul 20;8(3):233–47. doi: 10.1038/s41551-023-01067-5 (PMC10963274; doi:10.1038/s41551-023-01067-5)
Supplement: Supplementary file 2 — Reporting Summary [file 41551_2023_1067_MOESM2_ESM.pdf]

nature portfolio

Corresponding author(s): Christoph B. Messner, Markus Ralser

Last updated by author(s): Jun 5, 2023

Reporting Summary

Nature Portfolio wishes to improve the reproducibility of the work that we publish. This form provides structure for consistency and transparency in reporting. For further information on Nature Portfolio policies, see our Editorial Policies and the Editorial Policy Checklist.

Statistics

For all statistical analyses, confirm that the following items are present in the figure legend, table legend, main text, or Methods section.

n/a	Confirmed	
☐	☒	The exact sample size (*n*) for each experimental group/condition, given as a discrete number and unit of measurement
☐	☒	A statement on whether measurements were taken from distinct samples or whether the same sample was measured repeatedly
☐	☒	The statistical test(s) used AND whether they are one- or two-sided<br>*Only common tests should be described solely by name; describe more complex techniques in the Methods section.*
☒	☐	A description of all covariates tested
☐	☒	A description of any assumptions or corrections, such as tests of normality and adjustment for multiple comparisons
☐	☒	A full description of the statistical parameters including central tendency (e.g. means) or other basic estimates (e.g. regression coefficient) AND variation (e.g. standard deviation) or associated estimates of uncertainty (e.g. confidence intervals)
☐	☒	For null hypothesis testing, the test statistic (e.g. *F*, *t*, *r*) with confidence intervals, effect sizes, degrees of freedom and *P* value noted<br>*Give P values as exact values whenever suitable.*
☒	☐	For Bayesian analysis, information on the choice of priors and Markov chain Monte Carlo settings
☒	☐	For hierarchical and complex designs, identification of the appropriate level for tests and full reporting of outcomes
☒	☐	Estimates of effect sizes (e.g. Cohen's *d*, Pearson's *r*), indicating how they were calculated

Our web collection on statistics for biologists contains articles on many of the points above.

Software and code

Policy information about availability of computer code

Data collection	Analyst 1.8.1 TF (AB Sciex), ScanningSWATH RAW Converter (AB Sciex),<br>SCIEX OS 3.0.0.3363 & Waters ACQUITY Console Firmware 1.56 (MRM-HR data),<br>Tune 3.4 & Xcalibur 4.4 & SII 1.6 for Xcalibur (Orbitrap DDA data).
Data analysis	DIA-NN 1.8, python 3.8.8, OxoScan-MS associated library (see https://github.com/ehwmatt/OxoScan-MS), R 4.2.2, RStudio 1.2.5019, tidyverse 1.3.2, ggplot2 3.4.0, limma 3.54.1, EnvStats 2.7.0, ComplexHeatmap 2.14.0, Byonic 4.1.5, MSFragger-Glyco 3.7, FragPipe 18.0.<br><br>All custom code (OxoScan Python functions/Jupyter notebooks and R scripts for analysis and for reproducing all figures) and OxoScan-MS processed data for IgG, spike-in experiment and the COVID-19 cohort are freely available at https://github.com/ehwmatt/OxoScan-MS. Code with all accompanying processed data is also available at Zenodo (DOI: https://doi.org/10.5281/zenodo.8015483).

For manuscripts utilizing custom algorithms or software that are central to the research but not yet described in published literature, software must be made available to editors and reviewers. We strongly encourage code deposition in a community repository (e.g. GitHub). See the Nature Portfolio guidelines for submitting code & software for further information.

Data

Policy information about availability of data

All manuscripts must include a data availability statement. This statement should provide the following information, where applicable:

- Accession codes, unique identifiers, or web links for publicly available datasets
- A description of any restrictions on data availability
- For clinical datasets or third party data, please ensure that the statement adheres to our policy

Raw MS data (OxoScan-MS, DDA and MRM-HR), extracted oxonium ion .txt files from DIA-NN and OxoScan-MS processed outputs are available via MassIVE on ProteomeXchange (accession number: PXD034172). OxoScan-MS (Scanning SWATH) data can be opened in PeakView (AB Sciex) with a suitable license, and via Skyline. Source data for the figures in this study are available in figshare with the identifier https://doi.org/10.6084/m9.figshare.c.6677135.v1 (ref. 98). All processed data and accompanying scripts are also available from Zenodo at https://doi.org/10.5281/zenodo.8015483.

Research involving human participants, their data, or biological material

Policy information about studies with human participants or human data. See also policy information about sex, gender (identity/presentation), and sexual orientation and race, ethnicity and racism.

Reporting on sex and gender	Information on biological sex was derived from self-reporting, and not considered in our analysis.
Reporting on race, ethnicity, or other socially relevant groupings	Information on race or ethnicity or any other socially relevant grouping was not collected and thus not considered in our analysis.
Population characteristics	30 COVID-19 patients and 15 healthy individuals were included in the study. 47% (21/45) were female and 53% (24/45) were male. The median age was 50 (range 21–86). The severity was graded according to the WHO ordinal outcome scale of clinical improvement, with 15 with grade 0, 10 with grade 3, 4 with grade 4, 3 with grade 5, 3 with grade 6, and 10 with grade 7.
Recruitment	Sampling was performed as part of the Pa-COVID-19 study, a prospective observational cohort study assessing pathophysiology and clinical characteristics of patients with COVID-19 at Charité Universitätsmedizin Berlin (Kurth et al. Infection 2020). All patients with SARS-CoV-2 infection proven by positive PCR from respiratory specimens and willing to provide written informed consent were eligible for inclusion. Exclusion criteria were refusal to participate in the clinical study by the patient or legal representative, or clinical conditions that did not allow for blood sampling. The patients were hospitalized at Charité in Berlin between 1st and 26th of March 2020.
Ethics oversight	The Pa-COVID-19 study was carried out according to the Declaration of Helsinki, and the principles of Good Clinical Practice (ICH 1996) where applicable. The study was approved by the ethics committee of Charité-Universitätsmedizin Berlin (EA2/066/20).

Note that full information on the approval of the study protocol must also be provided in the manuscript.

Field-specific reporting

Please select the one below that is the best fit for your research. If you are not sure, read the appropriate sections before making your selection.

☒ Life sciences ☐ Behavioural & social sciences ☐ Ecological, evolutionary & environmental sciences

For a reference copy of the document with all sections, see nature.com/documents/nr-reporting-summary-flat.pdf

Life sciences study design

All studies must disclose on these points even when the disclosure is negative.

Sample size	Sample sizes were not predetermined on the basis of statistical methods.<br><br>For the proof-of-principle IgG experiments, a single replicate was used, as multiple comparisons (e.g. RT shifts for multiple IgG subclasses) could be observed in single samples.<br><br>Controlling the specificity of OxoScan with human plasma +/- deglycosylation used one replicate each, as the deglycosylation served as a strong negative control (that is, a very strong effect size).<br><br>Repeatability of glycopeptide quantitation with human plasma used 2 replicates.<br><br>Quantitative performance assessment via E. coli spike-in to human serum was measured as single replica per dilution.<br><br>Each sample of the COVID-19 cohort was produced and measured in OxoScan-MS mode in triplicate.<br><br>Glycopeptide ID from two pooled plasma samples (healthy and ill) by Orbitrap MS used one replica each.

	Validation by MRM-HR used single replicate injections of a different sample preparation starting from the same original patient plasma samples.
Data exclusions	No data were excluded.
Replication	Benchmarks were acquired in triplicates. COVID-19 cohort samples were prepared and measured as triplicates in OxoScan-MS mode, followed by validation by MRM-HR on another LC-MS platform (Waters M-Class + ZenoTOF 7600), in another lab. The findings were successfully reproduced.
Randomization	Samples were block-randomized whenever possible. The COVID-19 cohort samples were randomized.
Blinding	Measurements and analysis were not blinded, as there was no observer bias expected in this technical study.

Reporting for specific materials, systems and methods

We require information from authors about some types of materials, experimental systems and methods used in many studies. Here, indicate whether each material, system or method listed is relevant to your study. If you are not sure if a list item applies to your research, read the appropriate section before selecting a response.

Materials & experimental systems

n/a	Involved in the study
☒	☐ Antibodies
☒	☐ Eukaryotic cell lines
☒	☐ Palaeontology and archaeology
☒	☐ Animals and other organisms
☒	☐ Clinical data
☒	☐ Dual use research of concern
☒	☐ Plants

Methods

n/a	Involved in the study
☒	☐ ChIP-seq
☒	☐ Flow cytometry
☒	☐ MRI-based neuroimaging
